# A Taxicab geometry quantification system to evaluate the performance of in silico methods: a case study on adenosine receptors ligands

**DOI:** 10.1007/s10822-020-00301-5

**Published:** 2020-02-28

**Authors:** Kamil J. Kuder, Ilona Michalik, Katarzyna Kieć-Kononowicz, Peter Kolb

**Affiliations:** 1 Department of Technology and Biotechnology of Drugs, Jagiellonian University Medical College, Medyczna 9, 30-688 Kraków, Poland; 2 Department of Pharmaceutical Chemistry, Phillips-University, Marbacher Weg 6, 35037 Marburg, Germany

**Keywords:** Adenosine receptors, A_1_AR, A_2A_AR, A_3_AR GPCR, Homology modeling, Taxicab geometry, CBD

## Abstract

Among still comparatively few G protein-coupled receptors, the adenosine A_2A_ receptor has been co-crystallized with several ligands, agonists as well as antagonists. It can thus serve as a template with a well-described orthosteric ligand binding region for adenosine receptors. As not all subtypes have been crystallized yet, and in order to investigate the usability of homology models in this context, multiple adenosine A_1_ receptor (A_1_AR) homology models had been previously obtained and a library of lead-like compounds had been docked. As a result, a number of potent and one selective ligand toward the intended target have been identified. However, in in vitro experimental verification studies, many ligands also bound to the A_2A_AR and the A_3_AR subtypes. In this work we asked the question whether a classification of the ligands according to their selectivity was possible based on docking scores. Therefore, we built an A_3_AR homology model and docked all previously found ligands to all three receptor subtypes. As a metric, we employed an in vitro/in silico selectivity ranking system based on taxicab geometry and obtained a classification model with reasonable separation. In the next step, the method was validated with an external library of, selective ligands with similarly good performance. This classification system might also be useful in further screens.

**Electronic supplementary material:**

The online version of this article (10.1007/s10822-020-00301-5) contains supplementary material, which is available to authorized users.

## Introduction

A particular focus of rational drug design is the selectivity of novel ligands, with the aim to reduce possible side effects. The computational prediction of binding patterns of small molecules against multiple proteins would thus be of considerable interest. An in silico method that has worked particularly well with G protein-coupled receptors (GPCRs) is docking [1]. With respect to binding patterns, docking of a set of either newly designed ligands or virtual screening database compounds to various subtypes of a proposed biological target might narrow the group of potential ligands to those that exclusively interact with the intended protein(s).

GPCRs cover ~ 3% of the human proteome and represent the largest superfamily of membrane receptors. Built of seven transmembrane helices, they mediate signals from the out- to the inside of cells by sensing different agents. Binding of these agents leads to conformational changes and intracellular signaling cascades. Thus, GPCRs play a crucial role, either directly or indirectly, in the treatment of various pathophysiological states, evidenced by the fact that they are the targets of 30–50% of marketed drugs [2]. On the other hand, although the numbers have been rapidly increasing, only 62 members of the large family of GPCRs have been revealed as crystal structures up to date [3, 4]. Based on the fact that the transmembrane region of all GPCRs is well conserved, and knowing that most of class A GPCRs’ ligand binding cavities are open toward the extracellular region [5], homology modeling provides a useful tool for structure-based ligand design. However, the accuracy of the models can be limited, mostly in the area of extra- and intracellular loops. The reason for that can be sought in highly variable loop sequences often corresponding to unaligned regions in sequence alignments, as well as their location at the solvent-exposed surface of proteins that result in higher conformational flexibility [6].

Adenosine is an important regulator for homeostasis of the brain, heart, kidney and other organs. It interacts with four different GPCRs classified as A_1_, A_2A_, A_2B_ and A_3_ subtypes. Selective interaction with adenosine receptor (AR) subtypes offers very broad therapeutic potential, including CNS disorders, regulation of electrophysiological properties of the heart, immune system and inflammatory diseases, cell growth, asthma, kidney failure and ischemic injuries [7]. Adenosine receptors’ ligands are currently being developed as promising agents for CNS disorders (Parkinson’s, Alzheimer’s, epilepsy, ischemia) [8]. Also, the adenosine A_2A_ receptor has been co-crystallized with several ligands, agonists as well as antagonists, and serves as a model AR with a well-defined orthosteric ligand binding region.

In order to investigate the usability of homology models for SBDD, multiple adenosine A_1_ receptor (A_1_AR) homology models have been previously obtained and a library of lead-like compounds has been docked [9]. As a result, a number of potent and a few selective ligands toward the intended target were found. However, in in vitro experimental verification studies many ligands also turned out to bind to A_2A_AR and A_3_AR. Therefore, the aim of this work was to build an A_3_AR homology model, generated on the basis of the evolutionarily closest homologous templates, and dock all previously used ligands to all three receptor subtypes. We wanted to investigate whether the experimentally obtained binding profiles [9] can be reproduced in silico*,* as well as to see how, instead of looking at individual compounds, the set of compounds is predicted within a given campaign. Although A_1_AR crystal structures have been published recently [10–12], in this study we used a series of homology models for the sake of consistency with our previous study. Likewise, an approach to construct a reliable in silico/in vitro correlation quantification system has been undertaken and its usability has been validated with an external library of highly selective ligands.

## Materials and methods

### Homology modeling

In order to find the most suitable protein template for the A_3_AR receptor model, its sequence was obtained from UniProt database [13] (sp_P33765) and used for a BLAST search using two online tools: SwissModel [14] and ProteinBlast (NCBI) [15]. In both cases, the default search modes to find the most similar PDB crystal structures were used. After comparison of the results, three templates were chosen: 3EML (2.60 Å, 39.86% identity) [16], 2YDV (2.60 Å, 42.6% identity) [17] and 3VG9 (2.70 Å, 43.34% identity) [18]. Template proteins were chosen according to their highest crystallographic resolution (as well as crystal structures availability at the time) among two independent BLAST search hits, in order to increase the chances to obtain a reliable model.

Protein structures were pre-processed using PyMOL [19]: ligands, co-crystallization agents (2YDV, 3EML), the lysozyme insertion instead of ICL3 (3EML) were removed. Protein sequences obtained in this way were aligned using the PROMALS 3D [20] online tool. The resulting alignment, after visual inspection (position of transmembrane domains, possible disulfide bridges) was used as an input for MODELLER [21, 22].

Each of the 10 output models was then aligned to the 3EML crystal structure and carefully inspected visually using UCSF Chimera [23]. In particular, the orientation of the side chain of ASN250^6.55^ (superscript numbers denote Ballesteros–Weinstein numbers [24]) and other binding pocket amino acids was investigated and their possible, acceptable rotamers (according to the Dunbrack library [25]) were ascertained. Reasoning was supported by means of mutagenesis data [3, 4]. Similarly, we inspected the transmembrane domains to avoid gaps, obvious steric clashes, unnatural side chain amino acid folding, as well as a preservation of the disulfide bonds between CYS83^3.25^–CYS 166^45.50^.

### Known ligand database preparation

The next step was to test the enrichment of ligands over non-binders in the orthosteric binding pockets of the selected models. For this purpose, two sets of ligands were obtained from the ChEMBL database [26]. The “ligands” set consisted of approx. 1500 molecules described as A_3_AR ligands with a *K*_*i*_ ≤ 100 nM. Second, the decoy set consisted of approx. 800 molecules, tested against the A_3_AR and described as inactive for this target. Structures of both sets of ligands were obtained from the ZINC database [27] by searching for corresponding ZINC IDs for all of the ligands extracted from ChEMBL. High quality 3D conformer ensembles of both sets were obtained using the OMEGA module [28, 29] of the OEDocking software package (maximum number of conformers = 100; RMS = 0.5).

### Model refinement

The final A_3_AR homology model used in this study was obtained through the refinement process, using three different, consecutive strategies.

#### Strategy 1

As a reference ligand for docking, co-crystallized within the 3EML structure, the ligand ZM241385 was placed in the A_3_AR receptor models after their alignment to the 3EML structure, making sure that the hydrogen bonds with ASN250^6.55^ were formed. Two sets of ligands were then docked to the prepared receptor homology models using the HYBRID module (one pose per ligand, max. hitlist size 500 molecules), implemented in the OEDocking Software [30–33]. After docking, the top 500 poses were inspected visually, and receiver operator characteristic (ROC) curves were generated along with calculations of the area under the curve (AUC), using an in-house script.

Docked ligands were minimized using the SZYBKI module (OEDocking) [34] and the homology model of the protein (model 1) was minimized (with ligand present in the binding site) using CHARMM [35]. As several unfavorable energy poses and similar docking behavior was observed for the set of tested ligands, another modeling approach was then undertaken.

#### Strategy 2

Due to the fact that ZM241385 appears to be inactive towards A_3_AR and therefore might unduly bias the shape of the binding pocket during modeling, in a second round of modeling the previously identified ligand ZINC12533962, which is potent and selective towards A_3_AR (A_3_AR *K*_i_ = 40 nM) was placed manually in the crystal structure of 3EML instead, retaining similar ligand-receptor interactions. As the conformation of the ligand, the previously obtained pose from docking to the A_1_AR [9] was used. The protein conformation prepared this way served as a template using the same input alignment for MODELLER as for model 1, excluding the 2YDV and 3VG9 X-ray structures and including the ligand and its position during modeling.

From the output of ten models, the best scoring one (according to Modeller’s scoring functions; model 2) was chosen for evaluation.

#### Strategy 3

**Fig. 1:**
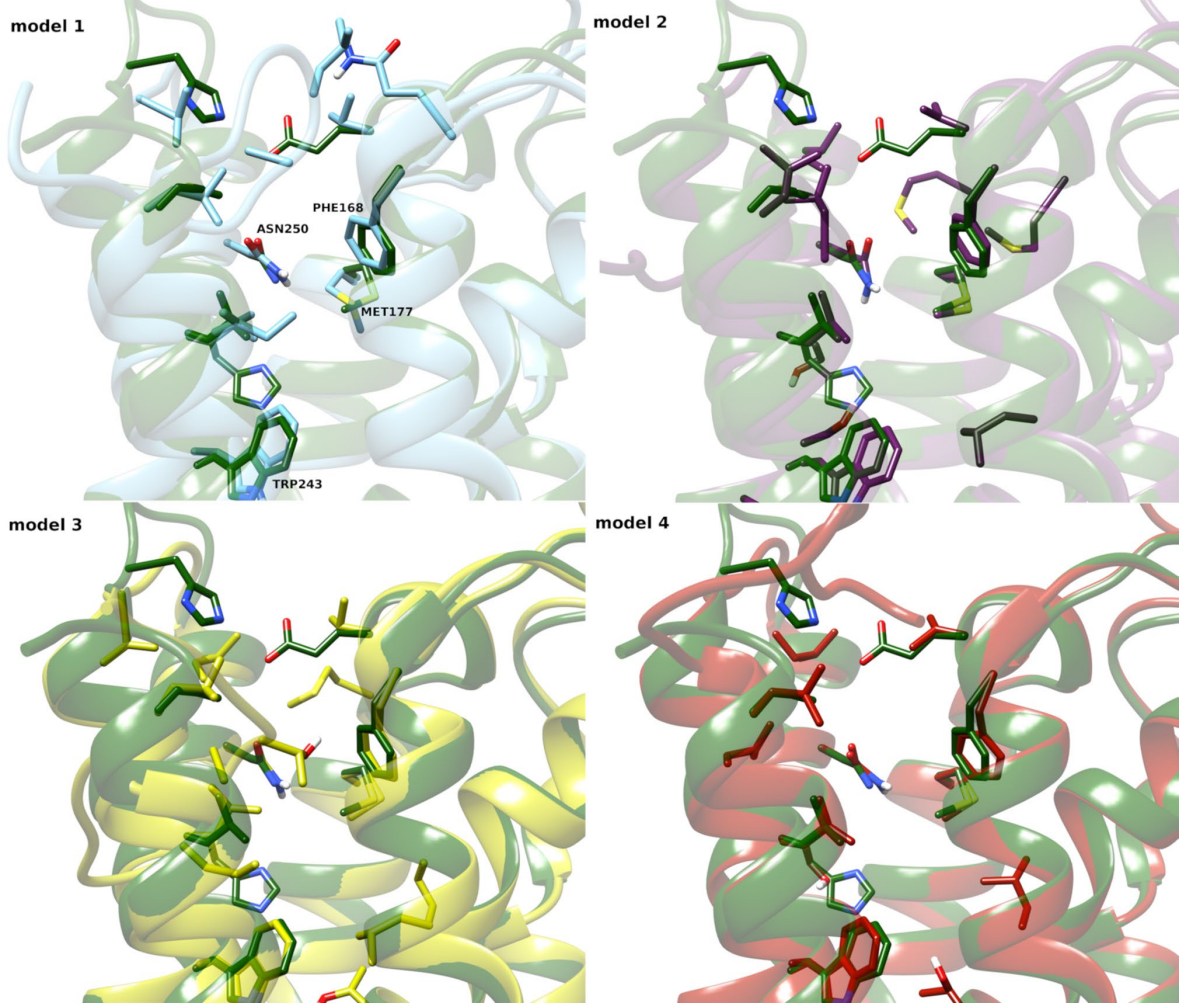
The four obtained A_3_AR homology models (1–4, detailed information can be found in Model refinement section; left to right, upper and lower panel). Binding pocket residues were depicted as thick sticks (only labeled in model 1 panel). The X-ray crystallographic structure of the template, A_2A_AR (3EML), is shown in dark green

Due to possible steric clashes between the ligand and TRP243^6.48^ in the bottom part as well as PHE168^45.52^ (ECL2) at the top of the binding pocket in model 2, again the position of ZINC12533962 in 3EML structure was corrected manually and the thus prepared receptor served again as a template for MODELLER. The resulting model (model 3) showed also potential steric clashes between the ligand and PHE168^45.52^, thus the ligand position in the template protein was again corrected, and the protein was remodeled. To the output model (model 4), after visual inspection, the set of actives and decoys was docked. This model (4), without further refinement and minimization, was chosen for all further docking studies. ECL2 was not remodeled, as it aligned well with the reference 3EML structure. Binding pockets of all four homology models obtained are presented in Fig. 1.

### Docking

In order to see whether the experimentally obtained binding profiles can be reproduced in silico, the next step involved docking of the previously described set of 39 ligands (test set) [9] to all three receptor subtypes (four A_1_AR homology models [9], the crystal structure of 3EML for A_2A_AR and the A_3_AR homology model). All ligands were prepared according to the same procedure described herein for the “binders/decoys” sets and docked to all receptors using the HYBRID module. As HYBRID docks multiconformer molecules into receptor-ligand complexes using an exhaustive search that systematically samples rotations and translations of each conformer of the ligand within the active site (defined by the “bound” ligand), no docking grid/sphere was set beforehand. For all of the docked sets, the preservation of a hydrogen bond with ASN^6.55^ as well as the orientation of the ligands in the binding pockets was inspected visually.

### In silico screening evaluation

For the quantification of the in silico/in vitro correlation, and, quite literally, to check how far from each other the results of those screenings are, an approach incorporating Taxicab geometry (City Block Distance, CBD) [36] and a traffic light system was utilized. Instead of the usual distance in Euclidean geometry, Taxicab geometry defines a new metric in which the distance between two points (*d*_1_) is the sum of the absolute differences of their Cartesian coordinates (*p, q*).

$${d}_{1}\left(p,q\right)={\Vert p-q\Vert }_{1}=\sum\limits_{i=1}^{n}\left|{p}_{i}-{q}_{i}\right|$$

Among a variety of everyday life applications, CBD systems can also be used to assess the differences in discrete frequency distributions. In our study, instead of Cartesian coordinates, in vitro (*v* = *K*_i_) and in silico (*s* = docking/rescore score) values were used for calculations.

$${d}_{1}\left(v,s\right)={\Vert v-s\Vert }_{1}=\sum\limits_{i=1}^{n}\left|{v}_{i}-{s}_{i}\right|$$

**Fig. 2:**
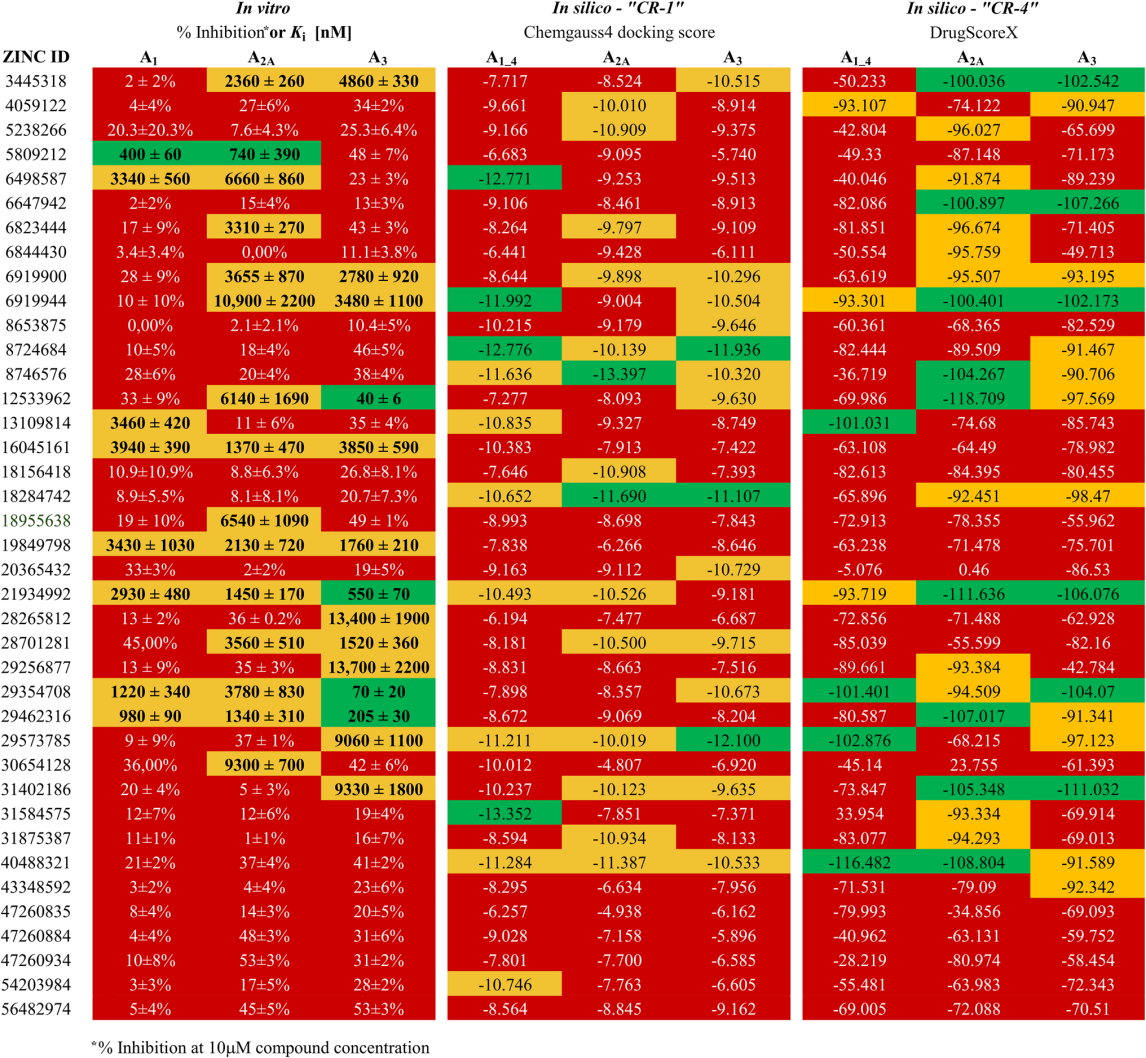
Distribution of CBD ranks for in vitro values (left panel), in silico, first docking run (“CR-1”, middle panel) and in silico second docking run with following rescoring (“CR-4”, right panel). Rank colors are assigned as stated in text. Detailed binding data can be found in [9]

So as to organize the results of both, the in vitro and in silico screenings, the results were in a first instance classified empirically (Fig. 2), according to the following key:

- for in vitro values: green for *K*_*i*_ values in the range below 1000 nM, yellow for *K*_i_ values higher than 1000 nM, but still at a measurable level, red expresses no detectable binding.
- for in silico values: green expresses first 20% of the obtained docking score range, yellow next 20% of the obtained docking score range, red expresses the remaining 60% of docking score range (preliminary partitioning),

while the CBD values (0–2) were assigned to each color in the manner: 0 for red, 1 for yellow and 2 for green (CBD calculation run 1; *CR-1*). The division for in vitro data remained unchanged for further data development. However, it has been shown that the position of the ligand pose closest to the native pose is distributed rather randomly among all generated poses and ordered with respect to the docking score [37]. Hence, the docking results were rescored using DSX-Online and the color scheme was adapted as follows: green < − 100, − 100 < yellow < − 90, red > − 90 (Fig. 2; *CR-2*). The next step of proposed platform evaluation was re-docking of the whole set of ligands to all four adenosine A_1_ receptor models, using ZINC12533962 as a reference ligand instead of ZM241385, in order to obtain a fair comparison for the docking procedure. This has been done by overlaying the obtained A_3_AR homology model onto the backbone of the A_1_AR models and preserving the coordinates of ZM241385. Using the same data partitioning as in the preliminary calculations, a CBD value was calculated (*CR-3*). A rescoring procedure was incorporated as described above (*CR-4*). To eliminate potential boundary effects arising from the in vitro/in silico data partitioning, the system was changed to a binary distribution (*CR-5*–*CR-8* for each previous run respectively) for either active or nonactive for the biological target (CBD = 1 for previous greens and yellows, 0 for reds) and recalculated (Fig. 4). Moreover, in order to determine the relative CBD value (CBD_rel_ = CBD/CBD_max_), the maximal possible CBD (CBD_max_) values for each distribution were calculated. A CBD_rel_ of less than 1 indicates better-than-random performance.

### Method validation

In order to test the usability and versatility of the described method, a library of 88 selective ligands previously described by Katritch et al. [38] was used. This ligand database was prepared according to the same procedure described herein for the “binders/decoys” sets and docked to all receptors using the HYBRID module. Likewise, the same data partition system as described in “In silico screening evaluation” subsection was applied. However, due to the high affinity of the ligands, the second system was incorporated:

- for in vitro values: green for *K*_*i*_ values in the range below 100 nM, yellow for *K*_i_ values between 100 and 1000 nM, red expresses over 1000 nM or no detectable binding

Detailed information on the used set along with partitioning systems incorporated can be found in the Supplementary Material.

## Results

### Homology modeling

The homology model of the A_3_AR developed for this study exhibited good quality, as characterized by the fact that 97.2% residues are in the favored region of the Ramachandran plot.

As MODELLER outcome, ten models (#mo1–#mo10; Modeller output) were constructed using the input alignment. Models were characterized by relatively high DOPE (Discrete Optimized Protein Energy, atomic distance-dependent statistical function), GA341 (describing reliability of a model, derived from statistical potentials) scores and molpdf values (molecular PDF, Modeller objective function—the sum of all restrains). The three best-scoring A_3_AR models (denoted: #mo3, #mo9, #mo2) were used for further evaluation strategies based on two main criteria: AUC of binders/non-binders docking evaluation and visual inspection of the docked ligands. The most convincing binding modes, as well as an AUC = 0.776 were observed for model #mo3, which was consequently chosen for further studies, and denoted as model 1.

Despite its high enrichment and acceptable binding modes of the “active” set of ligands, we have to note that possible steric clashes between ligands and amino acids were observed, as well as narrowing of the bottom part of binding pocket, indicating a low quality model that might lead to false results Therefore another modeling approach (denoted as Strategy 2) was undertaken.

Model **2** (molpdf: 1823.76904, DOPE score − 41,914.38281) was obtained as a result of strategy 2**,** by placing the highly potent and selective (A_3_AR *K*_i_ = 40 nM) triazine-based molecule ZINC12533962, into the binding pocket instead of ZM241385, the ligand inactive for A_3_AR, but co-crystallized within the 3EML structure, used again as a template. Since ZINC12533962 was a hit derived from the previous study [9] its selection for purpose of this study was straightforward. Models 3 (molpdf: 3149.86035, DOPE score: − 41,785.51562) and 4 (molpdf: 1593.80505, DOPE score: − 41,855.65625; used further in docking/selectivity studies) were the results of strategy 3. This entailed modeling after manual corrections of the ZINC12533962 ligand in the template structure, in order to obtain models with high enrichment ratios and possibly no steric clashes. The final model 4 used for the docking studies was characterized by a QMEAN score of 0.512 [39, 40], and 97.2% residues in the favored region according to the Ramachandran plot [41]. For all of the models, the orientation of the side chains in the binding side and the preservation of disulfide bonds was also checked visually. Binding pockets of all four obtained homology models are presented in Fig. 1.

### Known ligand database docking and CBD system evaluation

**Fig. 3:**
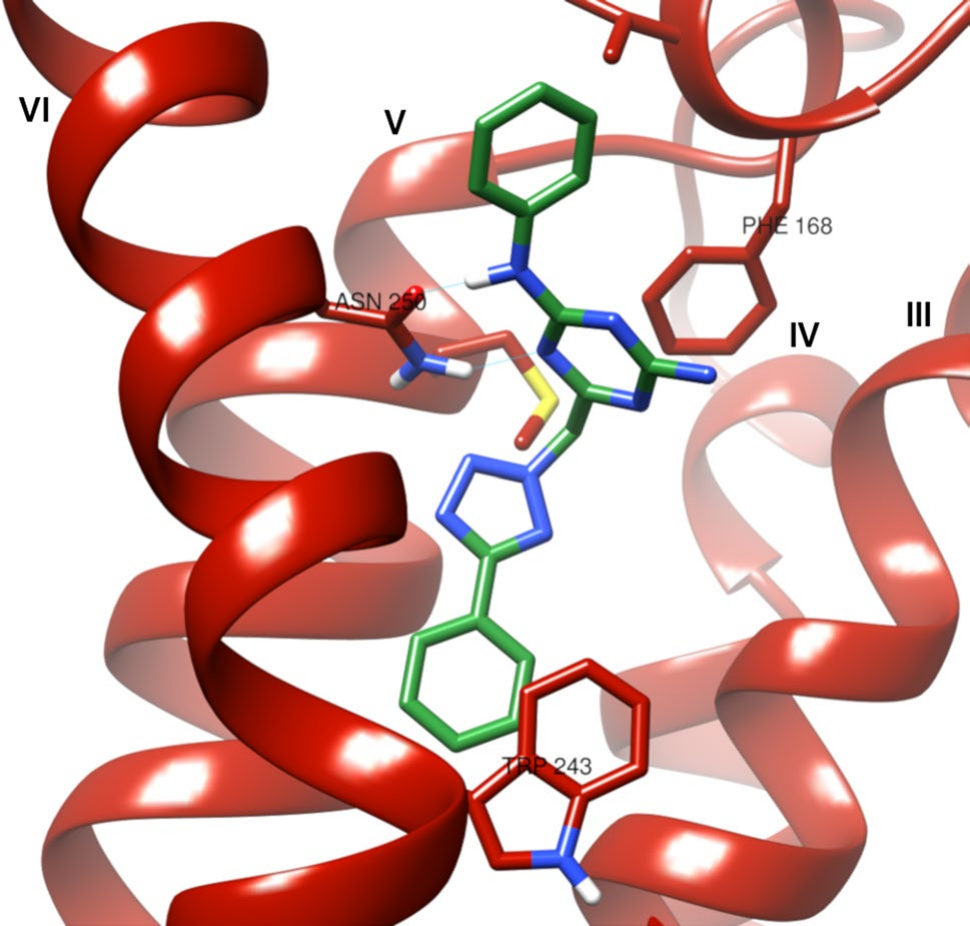
Calculated binding pose of ZINC 12533962 in adenosine A_3_ homology receptor (model 4) binding pocket. Blue lines indicate hydrogen bonds formed with ASN250^6.55^. Latin numbers indicate helices, helix VII was removed for better viewing purposes

As expected, most of the previously selected 39 test set ligands docked to the newly obtained A_3_AR homology model forming two hydrogen bonds with the key residue ASN250^6.55^ in the calculated poses. Also, for most in vitro* active* compounds, π–π stacking interactions between their (hetero)aromatic rings and PHE168^45.52^ were observed. Figure 3 depicts exemplary binding mode to the adenosine A_3_ receptor homology model 4.

In fact, in terms of productivity, our adenosine A_3_AR model gave an average true positive rate of 27 correct in silico/in vitro matches, out of 39 docked structures. A similar tendency was observed for all four used adenosine A_1_AR models, in contrast to, interestingly, a slightly lower score obtained using the crystal structure of the adenosine A_2A_ receptor. Also, exchange of the “original” ligand in the binding pocket of the A_1_AR models did not affect the overall productivity score. On the other hand, rescoring with DSX slightly increased the hit/non-hit ratio for the A_1_AR and A_2A_AR results.

**Fig. 4:**
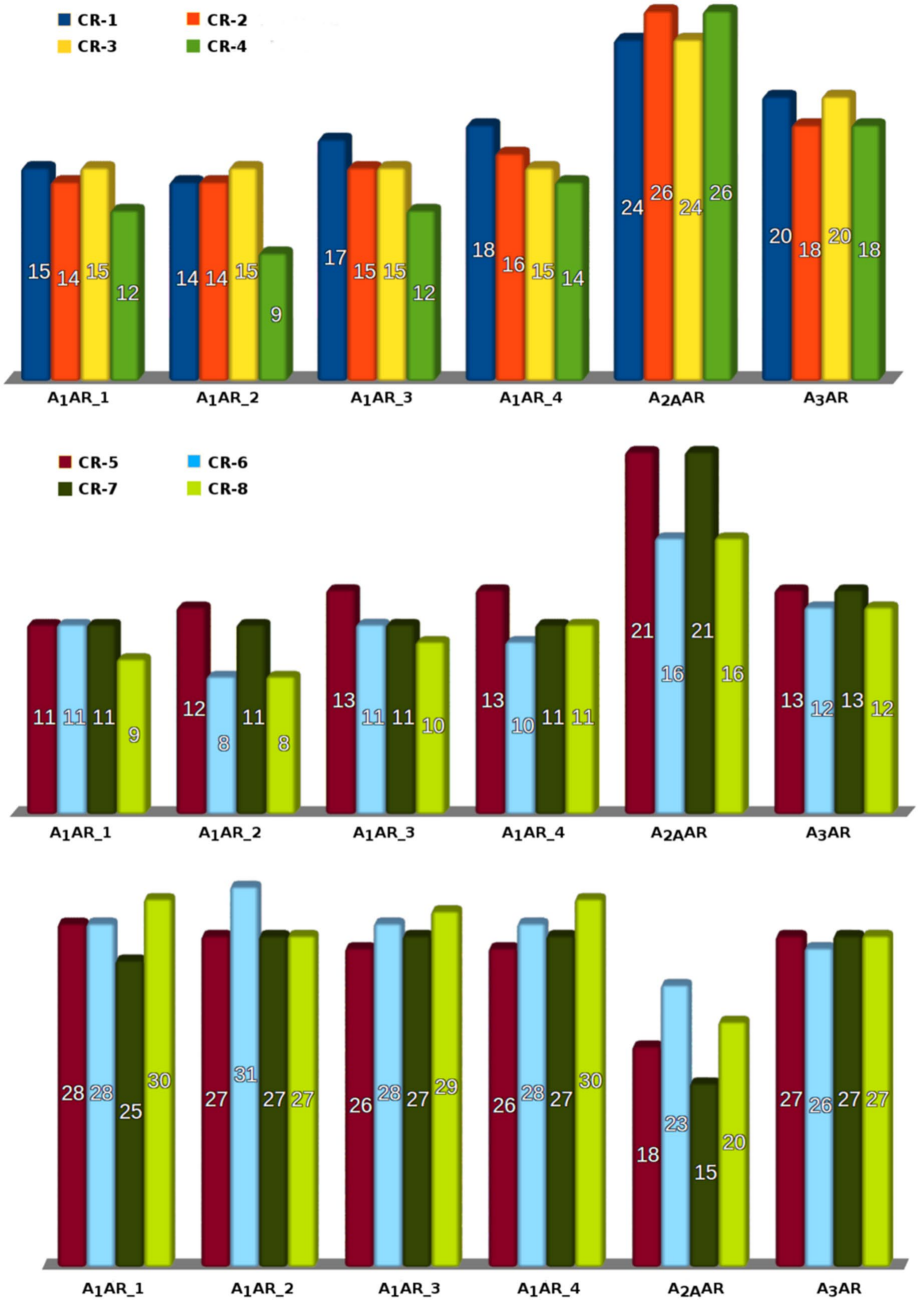
Upper panel: distribution of CBD values calculated in 0–1–2 mode for each receptor used; Middle panel: distribution of CBD values calculated in binary mode for each receptor; Lower panel: in vitro/in silico hit rate for all 6 used proteins (four A_1_AR models and one each for A_2A_AR and A_3_AR). Each column depicts one run according to the legend. For detailed information on proteins used, as well as “CR’s” please refer to “Materials and Methods”: Docking and In silico screening evaluation sections respectively

A similar influence was observed for CBD system incorporated in this study for selectivity prediction purposes (*CR-1* CBD = 61, avg = 1.56; *CR-3* CBD = 56, avg = 1.44). Rescoring, by increasing the productivity of models, resulted in slightly decreasing the CBD value in the first docking procedure (*CR-2* CBD = 60, avg = 1.53; *CR-4* CBD = 58, avg = 1.49). By changing the computational data partitioning to a binary system (either active or inactive, “greens & reds”, *CR-5*–*CR-8*) we were able to obtain CDB values of 46 for the docking (*CR-5*), and 37 for the rescoring procedures (*CR-8*). The same tendency was observed when calculating CBD values for particular targets (e.g. 13 vs. 12 for A_3_AR, Fig. 4) and relative CBD values as well. With CBD_max_ value of 172 for ‘0–1–2′ and 117 for binary distributions at given in vitro data, the CBD_rel_ values decreased from 0.36 (*CR-1*) to 0.32 (*CR-8*), proving the effectiveness of conducted calculations.

Overall, using the a priori data partitioning, starting from the average error of 1.56 blocks per compound (bpc), we were able to improve the CBD ranking method to an average error of 1.49 bpc. Binarization of the data partitioning allowed to decrease the distance even further, from average error of 1.18 bpc to 0.95 bpc.

### CBD system validation

The usability and versatility of the described CBD-based method has been assessed also with a set of reference compounds with strong selectivity, described by Katritch [38]. While the obtained CBD results of these validation runs (“VR”, for details please refer to Supplementary Material) were higher than for the test set, they still bore correlation to the *CR*’s. Starting from *VR-1* CBD resulted in avg. = 2.76 (when compared to 1.56 for test set), the method allowed for *VR-8* CBD avg. = 1.17. While the absolute values are somewhat different, a strong correlation with an R value of 0.96 (R^2^ = 0.92) between *CR* and *VR* runs can be determined. A tendency similar to the one for the test set—decreasing CBD_rel_ values for each run—was also observed. With CBD_max_ value of 476 for ‘0–1–2′ and 264 for binary distributions at given in vitro data, the CBD_rel_ values decreased from 0.51 (*VR-1*) to 0.39 (*VR-8*).

While the validation set expressed affinity for biological targets at much higher level than the test set, the sensitivity of method was increased, by exchanging the in vitro data partitioning system (Second Validation Run; *SVR*). With sensitivity set at *K*_i_ < 100 nM for greens and yellows for 100 nM < *K*_i_ < 1000 nM, CBD avg. obtained CBD values appeared slightly higher than those of *VR*’s (*CR*/*SVR* CBD R = 0.88, R^2^ = 0.77). However, the trend of decreasing CBD_rel_ values was maintained, and remained at the ~ 0.4 level.

In light of these validation results, we think that the ranking system herein presented can be used as an in silico/in vitro correlation quantification system independently of ligand selectivity.

## Discussion

Predicting the subtype selectivity of ligands to GPCRs using in silico methods still remains a challenge for modelers [37] for a number of receptors share a high degree of structural similarity among their subtypes. Adenosine receptors are no different indeed—all AR subtypes share core interactions within their conserved residues. These interactions include key strong hydrogen bonding with Asparagine in position 6.55, aromatic stacking with Phenylalanine in position 5.29 along with hydrophobic interactions with conserved Isoleucine and Leucine side chains in positions 7.39 and 6.51, respectively. Nonetheless, small mutations in overall highly homologous structures serve as key selectivity determinants for AR subtypes. In case of A_1_AR, that has a close similarity to A_2A_AR and A_2B_AR, a difference is caused by four mutations in the periphery of the binding pocket (e.g. position 7.35) and relatively shorter ECL2, which results in the formation of an additional hydrophobic sub-pocket in the loop region, allowing ligands to be more mobile in the binding pocket [42]. On the other hand, A_3_AR is the most disparate among ARs, having 10 (out of 20) unique amino acids in the binding pocket. The key difference is believed to be caused by exclusive Glutamate to Valine replacement in position 5.30 which plays an important role in ligand binding to for the remaining ARs [38].

Therefore, finding a ligand that is recognized by just one binding pocket and refused by all other subtypes heavily relies on the quality of protein models used for the studies, as well as the docking procedure, and data analysis. Up to date, a number of virtual screening approaches to find such ligands for adenosine receptors have been undertaken. Just to mention the latest few: Rodriguez et al. [43] were able to identify 9 A_2A_AR ligands out of 20 predicted while scanning a library of 6.7 million compounds, however none of them activated the target receptor. Later studies from this group incorporating virtual libraries allowed for the identification of two ligands targeting A_1_ and A_3_AR [44]. From the 63 structurally diverse ligands identified by VS by Tian et al. [45], 11 exhibited substantial activity against A_2A_AR in experimental tests, 2 of which with *K*_i_ in nanomolar level and good A_2A_/A_1_ selectivity. Last but not least, a non-typical, interesting incorporation of virtual screening in later stage of drug development, namely safety profiling, was described recently by Fan et al. [46].

Even though the selectivity of the compounds in the selected dataset was not extraordinary and we did not have the highest-affinity ligands known for the ARs in this set, for the sake of consistency with previous studies we used the same receptors and ligand sets. This also ensured data comparability. Moreover, we wanted to see how, instead of looking at individual compounds, the entire *set* of compounds is predicted—within a real-world campaign, one might not have a validated set of high-affinity ligands. From this study two main results emerged. First, our A_3_AR model is a reliable one in the sense that it is capable of recognizing the active ligands with high confidence (AUC = 0.844). This was also proven by accurate predictions for 27 out of the 39 ligands used in this study. The overall hit ratio is comparable with the A_1_AR homology models used in this study. Yet, in comparison to the A_1_AR models used for screening, the docking procedure involved only one A_3_AR homology receptor in a rigid state. Taking into account the high GPCR flexibility and the fact that only one out of a number of possible receptor conformations was used to obtain a relatively good hit ratio, it might be concluded that the obtained model can be further used for screening of larger libraries of compounds.

Second, the employed in silico ranking method based on Taxicab Geometry proved to be a useful metric to evaluate the performance of the in silico methods. The idea was to estimate and visualize how far from “exact” the screening results would be. As this study was the continuation of a previously described one [9] we already had the binding data in hand. Therefore, dividing the data into active and inactive compounds was the first step of applying the method.

At this point one has to deal with the uncertainties associated with experimental data. For this reason, and also to avoid classifying e.g. two compounds with 999 nM and 1001 nM affinity, respectively, as “active” and “inactive”, we introduced the “buffer” category yellow. The same applies for molecules with a percentage of inhibition close to 50%. In the docking part of our study, we chose to impose the set delimiters by percentage of the entire set, thus avoiding the issue of category-crossing error bars on e.g. ‘greens’ and ‘yellows’. By using a percentage-based splitting and focusing on the correlation of the entire set of molecules, the performance and assignment of an individual compound does not influence the overall performance to a large extent. Of course, in a prospective setting this percentage-based separation into categories is not obvious. Users might be guided by the commonly achieved hit rates in such cases, or, alternatively, by the capacity available for experimental testing.

On the other hand, dividing the computational docking data a priori was challenging. How to divide the docking results without being biased by the biological test data? This was even amplified when incorporating the third category, “moderately active”, to both sets of data to make the estimation more precise. However, even when juggling and correcting the screening data division, followed by redocking as well as rescoring of all of the poses, we were able to only get CBD values quite far from ideal. Nonetheless, comparing these values with an in-house script generating multiple 0–1–2 distributions, we were able to ascertain that the results were better than random. Further changing the data division to binary, either active or inactive, in fact yielded lower CBD values, resulting in an average error of less than 1 block per compound. Such a rank division seems satisfying, due to a high hit/no-hit ratio yield, clearly better than random, and should be accurate enough to be used also in a prosepctive setting. Again, using the same script generating multiple 0–1 distributions, results also appeared to be slightly better than random. The strength and effectiveness of these calculations were confirmed with relative CBD values calculated for each run, clearly proving their non-randomness.

As a further matter, for the purpose of method validation, we used a set of 88 highly affine and selective ligands. Albeit the obtained results appeared slightly higher than for the test set, they still prove the effectiveness of the method in both 0–1–2 and 0–1 distributions. The main reason for such performance might be the overall higher ligand affinity to the adenosine receptors. This issue was partly resolved by fine-tuning of the method, by increasing the sensitivity of in vitro data partitioning (eg. *VR-1* CBD avg. = 2.76 vs. *SVR-1* CBD avg. = 2.12). It also allowed us to show that such a sensitivity increase is not only possible, but also productive.

Nevertheless, docking to multiple receptors and ranking the data might be a challenge. First, four different homology models of one receptor (A_1_AR) generated only small differences in binding values, as the models only differ slightly from each other. Taking into account ranks from all four models seems like a sensible strategy, but care has to be taken not to bias the overall ranking just for this one target. Second, with each additional target and the set of screened ligands, the amount of data combinations increases, and ranks have to be divided carefully. Third, one must keep in mind that the assignment of docking ranks and scores to the different categories might still be influenced by the researcher deciding on the cutoffs.

Contemporary searches for novel GPCRs ligands rely heavily on docking of newly designed chemical compounds and virtual libraries to protein homology models and crystal structures. Still, the exact identification of hits and prediction of their selectivity profiles remains a challenge for computational chemists. The ranking system described herein might find its use in the search for selective compounds, but also those that are designed to act at more than one target. Despite its simplicity, it helps to condense a rather complex comparison into single numbers and cutoffs for classifiers.

## Electronic supplementary material

Below is the link to the electronic supplementary material.

Supplementary file 1 (RAR 2363 kb)

## Abbreviations

A_1_AR: Adenosine A_1_ receptor
A_2A_AR: Adenosine A_2A_ receptor
A_3_AR: Adenosine A_3_ receptor
AR(s): Adenosine receptor(s)
ASN: Asparagine
AUC: Area under the curve
BLAST: Basic local alignment search tool
bpc: Blocks per compound
CBD: City Block Distance
CNS: Central nervous system
CR: CBD calculation run
DOPE: Discrete optimized protein energy
DVR: Different partition CBD validation run
ECL: Extracellular loop
GPCRs: G protein-coupled receptors
ICL: Intracellular loop
PDB: Protein Data Bank/protein file format
PHE: Phenylalanine
QMEAN: Qualitative model energy analysis
RMS: Root mean square
ROC: Receiver-operator characteristic
SBDD: Structure-based drug design
TRP: Tryptophan
VR: CBD validation run
VS: Virtual screening
